# A Pilot Survey Study of Anterior Cruciate Ligament Injuries in Female University Athletes

**DOI:** 10.7759/cureus.62236

**Published:** 2024-06-12

**Authors:** Benjamin D Gompels, Holly Davis, Elizabeth Mainwaring, Georgia Tooth, Stephen McDonnell

**Affiliations:** 1 Department of Trauma and Orthopaedics, Addenbrooke's Hospital, Cambridge University Hospitals NHS Foundation Trust, Cambridge, GBR

**Keywords:** female athlete, orthopaedics surgery, orthopedic sports medicine, sports physiotherapy, knee injuries

## Abstract

Introduction

Female sports players are at increased risk of soft tissue knee injuries (STKIs) compared to their male counterparts. Injury prevention programs effectively reduce the incidence of anterior cruciate ligament (ACL) knee injuries. This pilot study, therefore, aimed to examine the prevalence, type, and management of STKIs within a population of female university sports players at the University of Cambridge. Additionally, this study aimed to examine the perceived risk of ACL injuries and knowledge of long-term complications, alongside participation and attitudes towards injury prevention programs.

Methodology

A survey was distributed to women’s university sports teams at the University of Cambridge. Information was gathered on participant demographics and sporting history. Relevant medical history, including joint laxity, connective tissue disorders, and previous knee injuries, was also collected. Participant involvement in and attitudes towards injury prevention programs were evaluated.

Results

Data from eighty-five participants (n = 85) were collected, all of whom were female. Forty-two percent of participants had sustained a previous knee injury, of which the majority (44%) were ACL injuries. In the ACL-injured group, 38% (n=6) had undergone ACL reconstructive surgery, 44% (n=7) had received only physiotherapy, and 19% (n=3) had received no form of treatment. Only 44% of these participants sustaining an ACL injury reported a return to the same level of post-injury sport. Seventy-two percent of respondents felt they were at increased risk of ACL injury compared to males. Most participants (87%) did not follow an injury prevention program, but 95% expressed a willingness to enroll in one.

Conclusions

This pilot study indicates that most knee injuries in female university athletes in this cohort at Cambridge University are ACL injuries, with a considerable number being managed conservatively. The low rate of return to pre-injury sporting levels highlights the significant impact of ACL injuries on athletic careers. This study demonstrates results similar to previous studies on the broader population. However, due to the pilot nature of the research and limited statistical power, the results should be interpreted with caution before transposing to the wider population. Further investigation is required into why many of these ACL-injured female athletes were managed conservatively and whether this finding is mirrored in their male counterparts. Despite recognizing their higher risk than males, participants displayed low engagement in injury prevention programs, indicating a gap between awareness and action. The willingness to participate in prevention programs suggests the potential for improved engagement through targeted interventions. Future research should focus on identifying and addressing specific barriers to participation in injury prevention programs and exploring the reasons behind the preference for conservative management of ACL injuries. Additionally, expanding the sample size and including a more diverse athletic population would enhance the generalizability of the findings.

## Introduction

The knee is the most injured joint within a sporting context. Knee injuries may account for 8-12% of all presentations to the emergency department, with 72% of knee injuries requiring surgical intervention being sports-related [1-3,4]. Anterior cruciate ligament (ACL) injuries are among the most common and significant knee ligament injuries, constituting almost half of all knee injuries, with a mean return-to-play time post-reconstruction being quoted at 12.2 months [5,6]. ACL tears are often career-ending; indeed, 20% do not return to any sport [7]. Female athletes are at an 8-9 times greater risk of ACL rupture than their male counterparts due to anatomical, hormonal, neuromuscular, and biomechanical differences [8-10].

The annual incidence of ACL injuries in 2017 was 71 per 100,000 individuals [11]. However, while the age and sex-adjusted incidence of ACL injuries appears to be declining in men in a 20-year longitudinal study from 1990 to 2010, it remains steady in women over this period [12]. One might speculate that the incidence of ACL injuries in females may rise with increasing participation in sports [13]. An estimated 15,000 ACL reconstructions are performed annually, but the figure is thought to be higher, with an estimated 12-fold increase over the past two decades [11,14,15].

Therefore, ACL injuries represent a substantial economic health burden, especially given the high prevalence of long-term sequelae, even despite ACL reconstructive surgery. Such long-term complications include early osteoarthritis, which affects 79% of patients, and a 1 in 5 risk of re-injury [16,17]. Indeed, with increasing media attention, there are growing calls for a focus on injury prevention, given the irreversible knee damage caused by ACL injuries [18].

Evidence from a recent meta-analysis showed that injury prevention programs can reduce ACL injury risk by 50% in all athletes and by 67% for non-contact ACL injuries in females [19]. However, despite the availability of such prevention programs, ACL injury rates in women appear to be on the increase [19].

The objectives of this pilot study are as follows: (1) To determine the prevalence and types of soft tissue knee injuries (STKIs) among female university athletes at the University of Cambridge; (2) To analyze the management strategies employed for these injuries within this population; (3) To assess the perceived risk of anterior cruciate ligament (ACL) injuries among female university sports players; (4) To evaluate the knowledge of long-term complications associated with ACL injuries among this population; and (5) To investigate participation in and attitudes towards injury prevention programs designed to mitigate the risk of ACL injuries.

## Materials and methods

The survey questions aimed to determine the prevalence and types of STKIs and analyze the management strategies employed for these injuries within this population. They also aimed to assess the perceived risk of ACL injuries among female university sports players and evaluate their knowledge of long-term complications associated with ACL injuries. Finally, the survey hoped to investigate participation in and attitudes towards injury prevention programs designed to mitigate the risk of ACL injuries.

Questions were developed based on known risk factors for STKIs by junior authors Gompels BD and David H. They were then reviewed and either approved or rejected by the senior author, consultant soft tissue knee surgeon McDonnell S. To our knowledge, this is the first study to focus on this particular series of questions in university athletes. The final questions focused on age, gender identity, height, weight, joint laxity, connective tissue disorders, previous knee injuries and treatments, and return-to-play time. Additionally, the survey gathered information on whether respondents perceived a heightened risk for women and if they were aware of any potential long-term effects following a knee injury. The questions included a variety of formats: multiple-choice, open-answer, and a Likert scale to allow respondents to estimate their perceived risk. It also asked respondents if they would follow a regular injury prevention program and how many hours per week they would be prepared to commit. There were 28 questions in total. Ethics, as per Cambridge University guidelines for survey studies, were followed; consent was obtained at the start of the survey from participants, and responses were stored securely. The complete survey can be found in Appendix 1.

The study included female athletes who had represented the University competitively in one of the following sports: rugby, football, netball, skiing, hockey, lacrosse, fencing, cricket, badminton, climbing, dance, volleyball, basketball, snowboarding, and tennis. Exclusion criteria included incomplete survey responses, athletes who had not competed at the university level, or athletes who were not of female sex.

The survey was distributed through the Central University Sports Network, specifically the Ospreys Committee, composed of each sport’s captains. It was open for three weeks from October 9 to October 30, 2023 and then closed, with 85 responses received. Out of the 15 surveyed teams, we received responses from players of 12 teams.

Statistical analysis was performed on the data collected on the perceived risk of a knee injury. The risk was categorized as more or less than five times more likely based on their perceived risk and then grouped into athletes who had sustained a knee injury and those who had not. A Fisher's exact test was performed using Prism version 9.5.0 (525).

## Results

Demographics and sporting history 

Eighty-five participants (n = 85) were enrolled, all of whom were female in both sex and gender. The age distribution ranged between 18-30 years old, with the majority (87%) being 18-23. Fifty-nine percent of participants had over eight years of sporting history and came from 11 sports teams. Most participants (82%) engaged in either rugby, football, netball, or hockey.

Relevant medical history 

A total of 15.3% (n=13) of participants self-reported having some hypermobility or joint laxity. No participants reported any history of connective tissue disorders.

Knee injuries and treatment 

**Figure 1:**
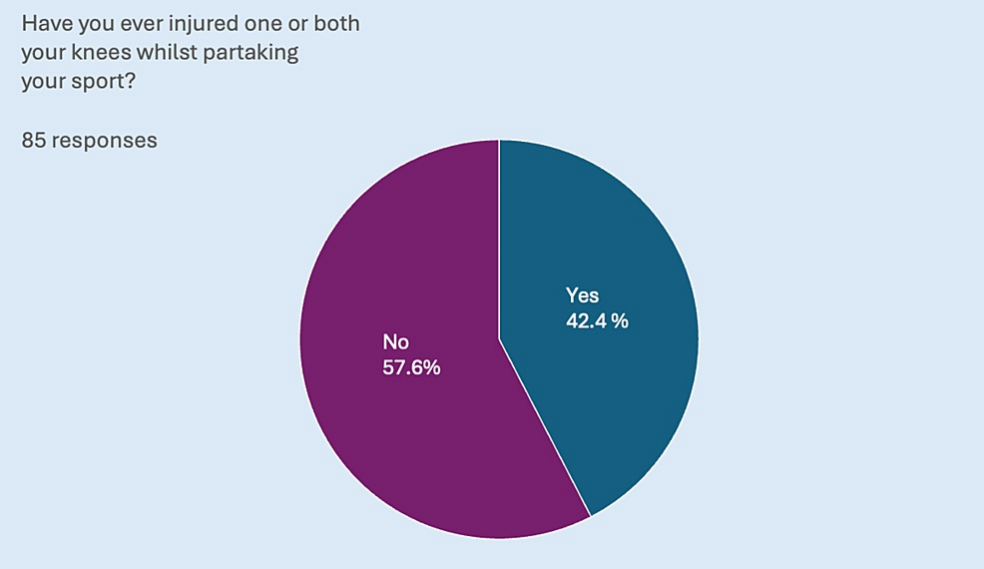
A pie chart showing that, out of a total of 85 respondents, over one-third have injured either one or both knees at some point.

Forty-two percent (n=36) of participants reported previous knee injuries (Figure 1), of which 44.4% (n=16) were ACL injuries. Among these participants, 38% (n=6) had undergone surgery, 44% (n=7) had received only physiotherapy, and 19% (n=3) had received no form of treatment. Of these ACL-injured participants, only 44% (n=7) successfully returned to the same level of sport post-injury.

Perception of ACL injury risk 

A total of 72% of participants felt they were at an increased risk of ACL injury compared to males. However, only 20% (n=17) identified the risk of developing osteoarthritis, and 16% (n=14) recognized the risk of re-injury. Athletes, whether they had injured their knee or not, were categorized by their perceived risk as more than five times or less than five times more likely to be at risk compared to their male counterparts of sustaining an injury. After performing Fisher's Exact test, there was no statistically significant difference in the odds ratio between the two groups (OR = 0.6710) (p-value = 0.591).

Awareness of and willingness to engage in prevention activities 

**Figure 2:**
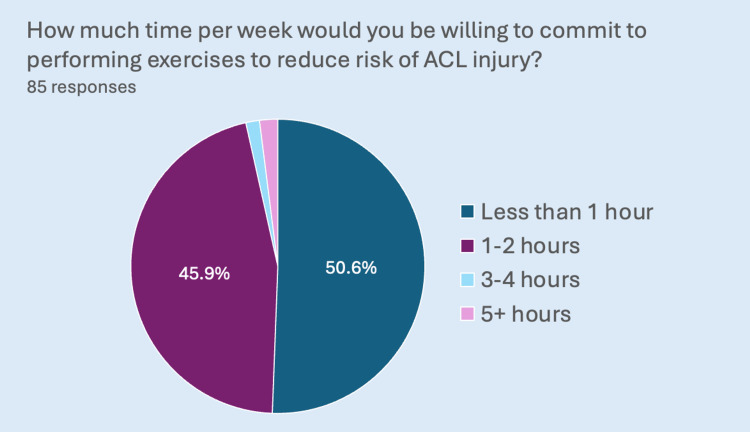
A pie chart detailing the number of hours respondents were willing to commit to performing exercises to reduce the risk of ACL injury. ACL: Anterior cruciate ligament.

A total of 87% of participants did not engage in ACL injury prevention programs or perform specific training or exercises to reduce ACL injury risk. Among the minority (13%) that engaged in injury prevention exercises, 46% (n=5) expressed dissatisfaction with the efficacy of these exercises in reducing their ACL injury risk. Of the total respondents (85), 95% (n=81) expressed a willingness to perform ACL injury risk-reducing exercises. Regarding the time commitment to ACL injury prevention programs, 51% of the total number of respondents (n=43) were willing to invest less than one hour a week, as observed in Figure 2.

## Discussion

Within our pool of participants, there was a high prevalence (42%) of soft tissue knee injuries, highlighting the significance of this injury type. Our study found that most of these STKIs were ACL injuries (44.4%), which was unsurprising, given that the ACL is the most injured soft tissue structure within the knee joint, constituting half of all knee injuries [5]. ACL injuries are among the most significant knee ligament injuries, with a mean return-to-play time post-reconstruction quoted at 12.2 months [6]. Indeed, only 44% of our ACL-injured participants reported successfully returning to the same level of sport post-injury, a finding that agrees with the literature, where ACL tears are often career-ending, with as many as 20% of individuals with reconstructed ACLs not returning to any sport at all [20]. Moreover, women have been shown to have an increased return-to-play time and a decreased return to sport compared to men [21].

Surprisingly, only 36% of our ACL-injured participants had undergone ACL reconstructive surgery; nearly a fifth of respondents who sustained an injury reported receiving no treatment. This mirrors findings in the literature, which identify gendered assumptions when planning care, often with negative consequences for women’s health [21]. Indeed, surgeons are more likely to offer ACL reconstruction surgery to men than to women [22]. Moreover, among those receiving ACL reconstruction, women have worse outcomes compared to men, with higher rates of revision surgery, future total knee arthroplasty, and lower patient-reported knee function. It is worth noting that the type of reconstruction or graft choice was not specified within these studies [23][24]. Further exploration of this research question could be done through qualitative data or additional quantitative analysis in future studies.

A total of 72% of our participants felt they were at increased risk of ACL injury compared to males. However, only 20% (n=17) identified the risk of developing osteoarthritis, and 16% (n=14) recognized the risk of re-injury. This finding suggests a disparity between the perceived risk of ACL injury and the actual risk. One might speculate that this disparity could be even more significant in the uninjured female sporting population. A recent meta-analysis has shown that injury prevention programs can reduce the risk of non-contact ACL knee injury by 67% in women [19]. Therefore, it was surprising that 87% of our participants did not engage in ACL injury prevention programs or perform specific training or exercises aimed at reducing ACL injury risk, especially given the high prevalence of previous ACL injuries in our participants. This may explain why Webster and Hewett observed that ACL injury rates appear to be increasing in women despite the availability of such prevention programs [19]. Future research must investigate the barriers to participation in such injury prevention programs. Barriers might include deficits in athlete and coach education, availability of injury prevention programs, or poor athlete compliance due to perceived time and financial costs [25]. Interestingly, over half of the participants (51%) would not be willing to invest more than an hour a week in an ACL injury prevention program despite the devastating nature of this injury.

In addition, athletes who had injured their knees did not perceive a higher risk of injury than respondents who had not injured their knees. This suggests that previous injury experience does not significantly alter risk perception. However, this result was statistically insignificant, so it must be interpreted cautiously. Previous studies focusing on the fear of re-injury found decreased levels of lower limb functionality, and fear of injury has also been identified as a risk factor for a new acute knee injury [26,27]. With a larger sample size and, therefore, more statistical power, the effect of knee injury on risk perception could be explored alongside qualitative research to further understand this psychological aspect of soft tissue knee injuries.

A significant limitation of this study was selection bias: one can speculate that participants were much more likely to respond to the survey if they had suffered a previous knee injury. This explains the disproportionately high prevalence of knee injuries (42%) within our participant pool, which is significantly higher than previously reported [28]. One would need to sample this population more randomly to better assess the prevalence of STKIs in this population. Additionally, our survey was only advertised to current sports team members. Therefore, we likely missed any women who have ceased playing sports following an STKI, questioning the validity of our return to play data.

Moreover, this study was limited by its small sample size and was heterogeneous regarding sports history, level of achievement, and sport type. This may limit the validity of our results and prevent further examination of injury patterns and risk perception according to the aforementioned factors relating to sport type, level, and years of play. To address these limitations, a future study should expand its sample size. Another limitation of this pilot survey was that it did not address the role of hormonal effects on the respondents, which has been identified as an independent risk factor in previous studies. Future studies of this nature could ask respondents if they were able to identify the stage of their menstrual cycle when they sustained the injury and whether they were on any medications that could alter their hormone balance, such as oral contraceptives or supplements. A final limitation of this study is the heterogeneity of participants. Respondents from different sports have varying injury risks and prevention practices, potentially affecting the study’s outcomes.

While our survey collected data on all types of STKI, most questions focused on ACL injuries, management, risk perception, and injury risk reduction. As a result, the scope of our study could have been expanded. However, this is partially justified, as ACL injuries are the most common form of STKI [5].

## Conclusions

This pilot study indicates a high prevalence (42%) of knee injuries among participants, with a significant portion (44.4%) being ACL injuries. The low rate of return to pre-injury sporting levels highlights the significant impact of ACL injuries on athletic careers. This study's findings align with existing literature, highlighting the frequency and impact of ACL injuries in female athletes. A considerable number of these injuries in this cohort were managed conservatively, with just over one-third managed surgically (38%). This trend toward conservative management requires further investigation to determine whether this finding is mirrored in their male counterparts. However, due to the pilot nature of the study and limited statistical power, the results should be interpreted with caution before being transposed to the wider population. Despite recognizing their higher risk compared to males, participants displayed low engagement in injury prevention programs, indicating a gap between awareness and action. The willingness to participate in prevention programs suggests the potential for improved engagement through targeted interventions. Despite an overwhelming majority expressing an interest in injury prevention programs, only a minority had enrolled. Future research should focus on identifying and addressing specific barriers to participation in injury prevention programs and exploring the reasons behind the preference for conservative management of ACL injuries. Additionally, expanding the sample size and including a more diverse athletic population would enhance the generalizability of the findings.

## Appendices

Appendix 1

ACL injuries in women partaking in sports at the university

This short survey aims to collect data on the occurrence and prevention of anterior cruciate ligament (ACL) injuries in women participating in sports at the university. It should take no more than 5 minutes to complete.

Responses will be used in an educational study on ACL injury and prevention and will be anonymized.

By completing the survey, you agree to allow your responses to be used in the study.

Section 1

What age are you?

Under 18

18-20

21-23

24-26

27-29

30+

**What is your gender identity?  Please select all that apply.** 

Female

Male

Transgender

Non Binary 

Is your gender identity the same as that assigned at birth?

Yes 

No

What is the primary sport that you partake in at the university?

Rugby

Football

Netball 

Volleyball

Skiing

Snowboarding

Tennis

Hockey

Lacrosse

Fencing 

Cricket 

Badminton 

Dance

Climbing

Other 

For how long have you participated in this sport?

Less than a year

1-2 years

3-4 years

5-6 years

7-8 years

8+ years 

At what sporting level do you currently participate?

Amateur (I am involved in organised sports and competitions at a non-professional level, for example, a college club)

Recreational (I participate in sports primarily for fun, exercise and social enjoyment with no formal competition)

Amateur (I am involved in organised sports and competitions at a non-professional level, for example, a college club)

University level (I am a university student-athlete and compete for my university, for example, as part of the 1st/2nd/3rd team)

Semi-professional/Professional level (I compete in sports at a semi-professional/professional level)

Which (if any) of the following movements do you consider to be involved in your sport?

Abrupt changes in direction

Abrupt changes in speed

Pivoting

Jumping and landing

Sidestepping

None of the above (please specify if able in 'other')

What is your approximate height (cm)?

What is your approximate weight in kilos?

Section 2: Previous Knee Injuries 

Have you ever injured one or both of your knees whilst partaking in your sport?

Yes 

No

Section 3: Previous Knee Injuries (Yes)

If yes, to your knowledge, was this injury to the anterior cruciate ligament (ACL)

Yes

No

How long ago was this injury (if you have had multiple injuries to the ACL, please answer based on the most recent injury)

Did you receive any support/rehabilitation for your ACL injury? Please specify 

Were you able to return to playing sports at the level you were at before injury?

Yes 

No

Not yet but I plan to 

Not yet and I do not plan to 

Section 4: Current Activities of Prevention?

Are you aware that women are at a greater risk of injuring their ACL in comparison to males?

Yes 

No

How much more likely do you think women injure their ACL in comparison to men?

0 times more likely 

1-2 times more likely 

3-4 times more likely 

5-6 times more likely 

7-8 times more likely 

More than 9 times more likely 

Not sure

Are you aware of any longer term risks of knee/ACL injury ? Please specify the risks you are aware of.

Are you aware of any evidence-based ACL injury prevention programmes? If so, please specify.

Do you currently undertake any programmes or other exercises/training to prevent ACL injury?

Section 5:  Specific Activities of Prevention 

What activities do you perform to prevent ACL injury? 

Do you feel that the exercises you perform are sufficient to reduce your risk of injury?

Yes 

No

Specific activities of prevention?

Section 6: Potential Activities of Prevention 

Would you consider performing (additional) exercises in order to reduce the risk of ACL injury?

Yes 

No

How much time per week would you be willing to commit to performing exercises to reduce risk of ACL injury?

Less than 1 hour 

1-2 hours

3-4 hours

5+ hours
